# Incidental diagnosis of right-to-left atrial shunt by computed tomography

**DOI:** 10.21542/gcsp.2022.17

**Published:** 2022-12-30

**Authors:** Yucel Colkesen

**Affiliations:** Erdem Hospital, Department of Cardiology, Bagcilar, Istanbul, Turkey

## Abstract

The existence and direction of an atrial shunt is normally diagnosed using echocardiography. A right-to-left atrial shunt, uncovered on routine computed tomography angiography, is presented. Transthoracic echocardiography verified the atrial shunt. TTE with intravenous agitated saline revealed the appearance of microbubbles in the left side of the heart. Atrial septal defects are a common cause of congenital heart diseases in adulthood. It may remain silent for decades because of the asymptomatic nature of the disease. Right-to-left atrial shunt is uncommon in patients with ASD.

## Description

**Figure 1.**
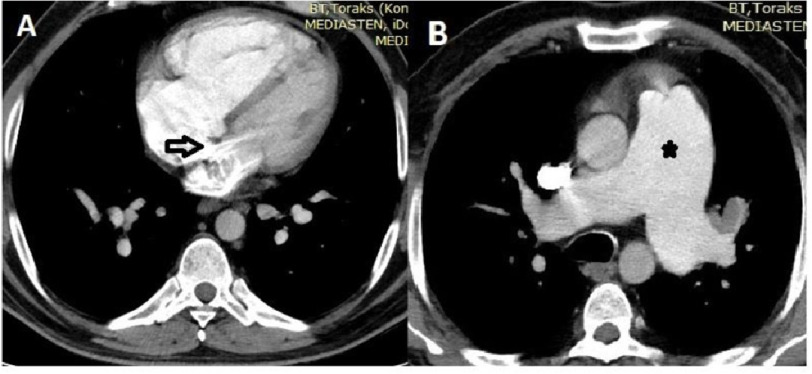
Four-chamber axial images on computed tomography illustrating contrast agent crossing the interatrial septum from right to left (arrow in panel A) and dilated pulmonary artery (asterisk in panel B).

**Figure 2.**
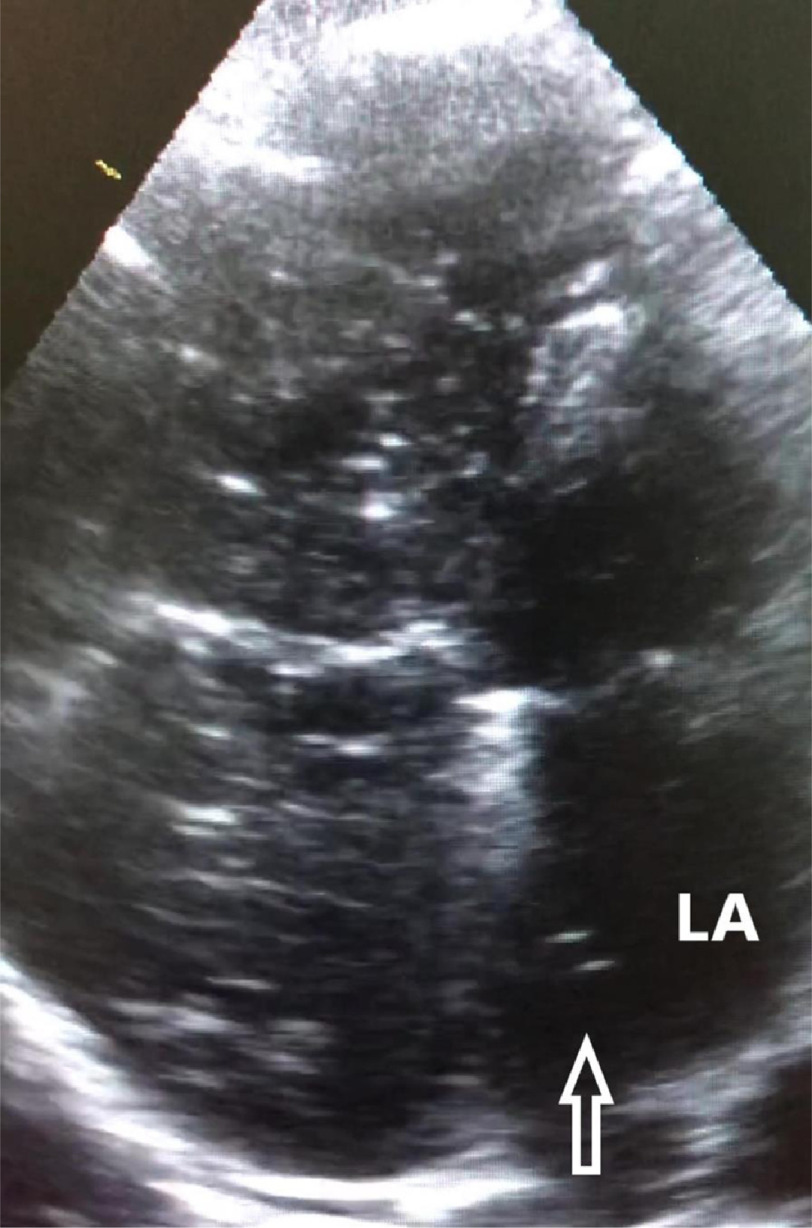
TTE using bubble contrast. Appearance of microbubbles (arrow) in left atrium (LA) in apical 4-chamber view on TTE is indicative of right to left atrial shunt.

A 54-year-old man, who was a heavy smoker, was referred to our hospital with progressive dyspnea on exertion, cough, and cardiomegaly on chest radiography. Physical examination revealed cyanosis of the nail bed and digital clubbing. His oxygen saturation was 89% on ambient air. Contrast-enhanced computed tomography was performed to determine the etiology of the hypoxemia. Four-chamber axial images illustrate the contrast agent crossing the interatrial septum from right to left (indicated by the arrow in Figure 1 panel A) and dilated pulmonary artery (asterisk in Figure 1 panel B). Transthoracic echocardiography was performed to verify the atrial shunt. The atrial septal defect was visualized, and the defect size was measured roughly using two-dimensional echocardiography. However, the transatrial jet could not be displayed with Doppler color flow imaging. TTE with intravenous agitated saline revealed microbubbles on the left side of the heart (Figure 2).

### What have we learned?

Atrial septal defects are a common cause of congenital heart diseases in adulthood. Late presentation of ASD can occur due to asymptomatic nature of the disease. The most common primary symptoms are exertional dyspnea and fatigue. The appearance of symptoms is not dependent on the shunt size, and symptoms may become evident at any age. In rare cases, a right-to-left atrial shunt may emerge and cause hypoxemia^1^. A transient rise in right atrial pressure above left atrial pressure may precipitate right-to-left shunting^2^. The patient was referred to a tertiary cardiac care center for left- and right-heart catheterization to determine pulmonary vascular resistance.

## Conflict of Interest

Author declares no conflict of interest.

### Funding

None.

### Ethical approval

I confirm that the appropriate ethics review has been followed.

### Consent

A signed informed consent has been obtained from the patient for publication of this case study and associated images.
